# Overnutrition and its associated factors among adult human immunodeficiency virus positive patients on antiretroviral therapy, Northwest, Ethiopia

**DOI:** 10.4314/ahs.v22i4.51

**Published:** 2022-12

**Authors:** Yihenew Sewale, Bitew Tefera Zewudie

**Affiliations:** 1 Department of Nursing, College of Health Science, Debre Berhan University, Debre Berhan, Ethiopia; 2 Department of Nursing, College of Health Science, Wolkite University, Wolkite, Ethiopia

**Keywords:** Overnutrition, ART, HIV

## Abstract

**Background:**

Anti-retroviral therapy was introduced to treat human immunodeficiency virus patients; comorbidities affecting individuals with human immunodeficiency virus-positive have changed dramatically, with increasing the prevalence of overnutrition. Overnutrition has increased from time to time in people living with the human immunodeficiency virus. However, there is scarce adequate documented evidence regarding nutrition on human immunodeficiency virus

**Objective:**

The study aimed to assess the magnitude of over nutrition and its associated factors among human immunodeficiency virus receiving antiretroviral therapy

**Methods:**

We used a cross-sectional study design to collect data from 422 participants from Debre Markos hospital. We used a systematic sampling technique to select the total number of participants. The outcomes of Data were entered, and coded using Epi-data version 4.1 and analysed using STATA Version 14.1. We performed a multivariable logistic regression model to identify determinants of over-nutrition at a p-value of less than 0.05.

**Results:**

The magnitude of overnutrition was 19.7% (95%CI: 14.6–25.4). Age group > 45 years (AOR: 3.18:95%CI: 1.09, 9.22), being farmer (AOR: 0.068, 95%CI (0.007, 0.611), family size greater than or equal to 4 (AOR: 3.18:95%CI (1.09–9.22), viral load less than 1000 copies/ml (AOR: 4.45 95%CI (1.69–11.76), and use of prophylaxis therapy (AOR: 2.67:95%CI (1.138–6.291) were significantly associated with over nutrition.

**Conclusions:**

In this study one-fifth of Human Immunodeficiency Virus/Acquired Immunodeficiency Virus patients had over nutrition. In this study, the magnitude of overnutrition is high associated with a viral load of fewer than 1000 copies/cell, age greater than 45, and having taken prophylaxis therapy. Therefore, education about lifestyle change, regular monitoring of weight, regular nutritional assessment, and intervention of the existed problems like doing regular exercise is highly recommended.

## Introduction

Overnutrition is a patient with a body mass index between 25 and 30 Kg/m^2^ and greater than 30 Kg/m^2^ 1. According to the World Health Organization (WHO), overnutrition remains “one of today's most blatantly visible-yet most neglected-public health problems”^2^. The prevalence of overnutrition across the world has increased by more than 200% since 1980 with nearly 2 billion adults estimated to be overnutrition in 2014^3^. The prevalence of overnutrition is an increasingly important public health concern in sub-Saharan Africa, especially in urban areas^4^. Anti-retroviral therapy was introduced to treat HIV infection, and comorbidities affecting individuals with HIV infection have changed dramatically, with increasing the prevalence of overnutrition^5^. Human immunodeficiency virus-infected individuals are seriously affected by nutrition^5^,^6^, and the prevalence of overnutrition is also rising^7^,^8^. Long-term ART is associated with a multitude of metabolic abnormalities of over nutrition similar to uninfected individuals, evidenced that a combination of treated HIV infection and excess adiposity may compound the risk for the development of comorbid conditions^9^. Overnutrition is a growing public health problem among HIV-positive people on ART worldwide. Increased weight and physical inactivity have been attributed to overnutrition among HIV-positive people on ART^10^. Overnutrition among people with ART has been characterized by a dorso-cervical fat pad and visceral fat accumulation that may increase the risk of cardiovascular diseases^11^.

Overnutrition has increased from time to time in people living with the human immunodeficiency virus with ART^12^,^13^. The study which is conducted in the USA, Atlanta, Maryland, Brazil, California, Tanzania, Zimbabwe, France showed that the prevalence of overnutrition was (46%)^14^ (42%)^15^ (49)^16^,(39.8%)^17^, (33%)^18^, (63%)^19^,(26.4%) 20, (27%)^21^ respectively. A study conducted in Ethiopia found that magnitude of overnutrition among HIV-positive patients with ART was 21%^22^.

Although responsible factors for over-nutrition are multi-factorial, several studies showed that being female, older age (greater than 45 years)^20^, ^21^, CD4+ lymphocyte count >350 cells/mm^3^ 18,22,23, being married^18^, WHO clinical stage III and IV and physical inactivity^22^, duration of HAART^14^ were factors associated with overnutrition in people living with HIV receiving antiretroviral therapy. In Ethiopia, the government designs different strategies to achieve sustainable development goal (SDG) #2. For example, national consolidated ART care and end hunger, achieve food security and improved nutrition and promote sustainable agriculture yielding marked success in reduction of HIV and over nutrition patient morbidity and mortality^24^, ^25^ respectively. Despite the seriousness of the problem, a study conducted to evaluate factors responsible for overnutrition among HIV-positive persons during the ART period remains largely unknown. Therefore, this study aimed to identify factors associated with over-nutrition and its magnitude. The results obtained from this study will improve the quality of care of clients in the study area and similar settings in Ethiopia.

## Methods and materials

### Study design, setting, and period

The study was conducted at Debre Markos Referral Hospital. The hospital is situated in Debre Markos Town and is located Northwest of Ethiopia 300 Km away from the capital city of Addis Ababa. It was built in 1957 to serve about 25,000 people and now it is serving more than 3.5 million people. A facility-based cross-sectional study design was employed from March 1 to May 1, 2020. We have recruited HIV/AIDS patients from one comprehensive specialized hospital rendering ART treatment services. An average of 3220 patients per month in the ART clinic have had fellow up.

### Inclusion and exclusion criteria

We included all HIV-positive adults who received ART and were available during the data collection period at the ART clinic. We excluded patients who were pregnant and developed over nutrition before initiating ART.

### Source of population, sample size determination, and sampling procedure

Of the total 3220 adult patients with HIV/AIDS at the ART clinic, 422 participants were used for data collection. We used a systematic random sampling technique to select the participants for an interview, anthropometric measurement, and clinical records review from the register. The sampling interval (kth) was determined by dividing the total HIV/AIDS positive patients of the hospital allocated sample size. k=N/n=k=3220/422≈8 The first sample was selected by simple random sampling from clinical records and every (kth) was selected for gathering information until the required sample was obtained from the study participant. Assuming 50%, prevalence of over nutrition, 5% precision, 95% confidence level, and 10% non-response rate. We calculated a sample size of 422 adult HIV/AIDS patients. 
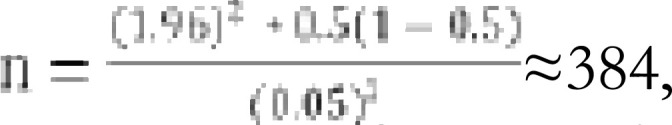
 after adding a 10% non-response rate, the final sample size was 422. Next, we used a proportional systematic random sampling was performed to select the eligible participants, who fulfilled the set inclusion criteria, from the total patients from daily attended patients.

### Study participants

All HIV-infected adults receiving ART have a follow-up in the ART clinic and are available during the data collection period.

### Outcome variable

The outcome of interest was over nutrition as measured using the body mass index (BMI), defined as the weight in kilograms divided by the square of the height in meters (kg/m^2^). The body weight was determined to the nearest 0.1 kg on a standing electronic digital scale and height was measured to the nearest 0.1 centimetres (cm). BMI was calculated for each participant and their nutritional status was categorized according to World Health Organization (WHO) standards. Accordingly, a participant was declared to have overnutrition if the BMI metric is between 25 and 30 Kg/m^2^ and a patient with a body mass index of greater than 30 Kg/m^2^ 1.

### Explanatory variables

Data were socio-demographic characteristics, including the age of the participants, sex, residence, occupation educational status family size, nutritional support, and counselling. Clinical-related factor information included CD4 count, viral load, ART adherence, WHO stage, OI, ART duration, ART regimen, prophylactic therapy, comorbidity diseases, and physical inactivity.

### Operational definitions

**Overnutrition:** a patient with a body mass index between 25 and 30 Kg/m^2^ and a patient with a body mass index of greater than 30 Kg/m^2^ 1.

**Adult:** a person whose age is ≥18 years old.

**Adherence to ART:** the recent adherence status of the adult to the ART is recorded as poor, fair, and good based on leftover pill count. Adherence is poor when an adult takes less than 85% of the dose, fair when he/she takes 85–94% of the dose, and good when he/she takes 95% and above of the dose^24^.

**Comorbid diseases:** are chronic diseases with a confirmed diagnosis of a disease other than HIV infection^24^.

**Opportunistic infections/diseases:** is the list of opportunistic diseases indicated in national comprehensive HIV prevention, care, and treatment^24^.

**Prophylaxis Therapy:** A co-trimoxazole/isoniazid preventive therapy that is implemented as an integral component of a package of HIV-related services based on an indication of prophylaxis^24^.

### Data collection instruments

For the proposed study three data collection instruments were used. Primary data were collected using a structured interviewer-administered questionnaire, body mass index has done using standardized and calibrated weight and height measuring scales, and secondary data were reviewed from medical records.

### Data collections and management

We used an interviewer-administered and structured questionnaire to collect the data. The questionnaires were adapted and modified into local context from previous works of literature^26^–^35^. FAO standard verbal questionnaires and a national comprehensive consolidated ART guideline checklist were also used^36^. To assure data quality, the tool was first prepared in English and then translated to Amharic and back to English. Besides, a principal investigator performed daily supervision. The two-day training was given for data collectors concerning the data collection tool and data collection process. We have done a pre-test on 10% of the total sample size at Finot Selam General Hospital, and the questionnaire was double-checked for completeness both by supervisors and data collectors. Data were collected at the time of ART care follow-up by interviewing all participants who attended the ART clinic. Additionally, we obtained clinical data by reviewing the chart. Four nurses screen the participants and collected data based on the inclusion criteria. Furthermore, we checked the collected data for completeness and consistency during the data management, storage, and analysis.

### Statistical Analysis

Data were entered into EPI DATA version 4.1, and analysis was performed using STATA version 14.1 statistical packages. Descriptive statistics, like frequency and percentage, were used depending on the nature of the variable. Binary logistic regression analysis was computed to assess the associations of the independent variables with over-nutrition. In the bivariable analysis, variables with a p-value of less than 0.25 were entered into the final model. In this analysis, a potential problem of multicollinearity was checked using the variance inflation factor (VIF) at the cut-off point of 10. Finally, in the multivariable binary logistic model, a p-value of <0.05 was considered to declare statistical significance with the corresponding 95% confidence interval. The goodness of fitness of the model was checked using the Hosmer-Lemeshow GOF test. The p-value of the Hosmer-Lemeshow GOF test of our model is 0.562 which confirms that the model is correctly specified.

## Results

### Socio-demographic characteristics of study participants

The response rate was 97.6% with more than half 240 (58.3%) of participants being females and the majority of 339 (82.2%) being from urban areas. The mean (±SD) age of study participants was 41.1 (±11.3) years. More than half of 250 (60.68%) participants had an age group of greater or equal to 45 years. Greater than one-third of 196 (47.6%) of participants were 1–12 grade, and more than two-thirds of 288(69.9%) got nutritional counsel and support. More than one-third of 144 (38.1%) participants had greater or equal to 4 family sizes (Table 1)

**Table 1:** Socio-demographic characteristics of the study participants at Debre Markos Compressive Specialized Hospital northwest, Ethiopia 2020

variables	Frequency (N)	Percent (%)
Sex		
Male	240	58.3
Female	172	41.7
Residence		
Rural	339	82.2
Urban	73	17.8
Age		
≥45 year	250	60.68
>45 year	162	31.32
Marital status		
Married	181	43.9
Divorced	97	23.5
Windowed	89	21.6
Others	45	10.9
Occupation		
Farmer	103	26
Merchants	69	16.7
Governmental worker	90	21.8
Private worker	81	19.7
Others	65	15.8
Educational status		
Unable to read and write	126	30.6
Able to read and write	36	8.7
1–12 grade	196	47.6
College/university	54	13.1

### Clinical related characteristics of participants

A majority (85.44%) of participants did not make regular physical activity, and less than one-fourth (22.6%) of participants were taking prophylaxis therapy. Approximately one-tenth of 40 (9.8%) of participants had greater than or equal to 1000 copies/ml of viral load, the majority of 291(70.9%) of participants had WHO stage III, and more than two-thirds of 286 (69.4%) of participants did not develop OI. One-fourth of 103 (25%) participants have taken a combination of protease inhibitors with nucleoside reverse transcriptase, more than one-fourth (29.6%) had less than 500 cell/µL CD4, and more than one-third (40%) of participants had received ART greater than or equal to 5 years (Table 2).

**Table 2:** Clinical-related characteristics of the study participants at Debre Markos Compressive Specialized Hospital, Northwest, Ethiopia, 2020

Variables	Frequency (N)	Percent (%)
Physical activity		
Yes	60	14.56
No	352	85.44
Prophylaxis therapy		
Yes	93	22.6
No	319	77.4
Viral load		
<1000 copies/ml	372	90.2
≥1000 copies/ml	40	9.8
WHO stage		
I	291	70.6
II	51	12.4
III	42	10.2
IV	28	6.8
Comorbidities		
Yes	24	2.3
No	388	97.8
OI		
Yes	126	30.6
No	286	69.4
ART regimen		
1e(TDF+3TC+EFV)	134	32.5
IJ(TDF+3TC+DTG)	157	38.1
2h(TDF+3TC+ATV/r) &2f(AZT+3TC+ATV/r) &2e(AZT+3TC+LPV/r) &ABC+3TC+ATV/r	103	25
Others	18	4.4

### The magnitude of overnutrition among HIV positive patients

The magnitude of overnutrition was 19.7% (95%CI: 14.6–25.4) among Adult HIV-positive patients attending the Art clinic.

### Associated factors with overnutrition among HIV positive patients

In bi-variable logistic regression analysis, variables (P-valve <0.25) of residence, age, occupation, education, marital status, family size, stage of WHO, Diabetes millets', kidney diseases, prophylaxis therapy, viral load, and duration of HAART were associated factors with over nutrition. In the final model, only five variables were statically significant associated factors with overnutrition. Accordingly, participants who were being farmers were 93.6% times less likely to develop over nutrition as compared to others (AOR: 0.068, 95%CI (0.007–0.611), and a participant who were age group > 45 years were 3.18 (AOR:3.18:95%CI (1.09–9.22) times more likely to develop over nutrition as compared to age group less than or equal to 45 years.

A participant with a family size greater than or equal to 4 was 2.49 (AOR: 2.49: 95%CI (1.32–4.67) times more likely to develop over nutrition compared with a family size 1–3. A participant who had a viral load of fewer than 1000 copies/ml was 4.45 (AOR: 4.45 95%CI (1.69–11.76) times more likely to develop over nutrition compared to their counterpart. Finally, Participant who had taken prophylaxis therapy was 2.67 (AOR: 2.67:95%CI (1.138–6.291) times more likely to develop over nutrition as compared to their counterpart (Table 3).

**Table 3:** The bi-variable and multivariable logistic regression analysis of associated factors of over nutrition at Debre Markos Compressive Specialized Hospital northwest, Ethiopia, 2020

Variable	Over nutrition		COR (95%)	AOR (95%)	p-value
				
	yes	No			
Age category					
≤45 years	35	215	1	1	
>45 years	40	122	3.51(1.56,9,78)	3.18(1.09,9.22)	0.03
Family size					
1–3	35	220	1	1	
≥4	40	117	1.86(1.10,3.16)	2.49(1.32,4.67)	0.004
Occupation					
Farmer	10	93	0.076(0.009,0.608)	0.06(0.07,0.61)	0.017
Merchants	19	50	1.235(0.536,2.847)	0.93(0.34,2.52)	0.899
Governmental	24	66	1.182(0.532,2.625)	0.77(0.26,2.25)	0.778
Private worker	10	71	0.458 (0.181,1.155)	0.43 (0.15,1.24)	0.436
Others	12	53	1	1	
Prophylaxis therapy					
Yes	9	84	2.435 (1.163,5.097)	2.67(1.13,6.291)	0.024
No	66	253	1	1	
Viral load					
<1000 copies/ml	64	308	1.82 (1.38,3.84)	4.45(1.68,11.75)	0.003
≥1000 copies/ml	11	29	1	1	

## Discussion

In general, this study has demonstrated over nutrition affects one in every five of the sample population, and factors such as older age, being farmer, family size greater than or equal to 4, viral load less than 1000 copies/ml, and use of prophylaxis therapy were significantly associated with over nutrition. Magnitude of overnutrition among HIV positive patients attending ART clinics of Debre Markos hospital was 19.7% (95%CI: 14.6–25.4). This finding is in line with the previous studies conducted in Zimbabwe (26.4%)^20^, France (27%)^21^, and Ethiopia (21%)^22^. However, this study's findings were lower than the studies conducted in the United State of America (46%)^14^, Atlanta (42%) 15, Maryland 49)^16^, Brazil,(39.8%)^17^, California (33%)^19^ Tanzania (63%)^18^. The above disparity between studies could be explained by the study settings, ART duration, and regimens, culture of the feeding of the study participants.

This study showed that older age, being a farmer, family size greater/equal to 4, use of prophylaxis therapy, and viral load less than 1000 were statically significant associated factors with over nutrition. This study showed that older age was statically associated with overnutrition. These findings were consistent with studies reported from Ethiopia^22^, France^21^, Atlanta^15^, Tanzania^18^, California^19^, and Zimbabwe^20^. This might be due to the paradox curves of overnutrition reaching the maximum point in the mid-50s and then declining. This study found that family sizes greater and equal to 4 were statically significant associated factors with overnutrition.

This study found that family sizes greater and equal to 4 were statically significant associated factors with overnutrition. This finding is not consistent with other study reports^5^, ^15^, ^16^, ^22^. The main justification could be most of the patients had taken prophylaxis therapy, had a viral load of fewer than 1000 copies/cell, and were physically inactive. Besides this, good availability, accessibility, stability, and utilization of food and nutrition in the house.

Participants who had a viral load of fewer than 1000 copies/ml were at high risk for developing over nutrition compared with those who had greater than and equal to 1000 copies/ml. this finding is to align with the study conducted in Boston, USA^37^. It is plausible that leptin may be involved in the pathway of overnutrition to immune functioning, and it is an adipocyte-derived hormone that influences body weight^38^. The possible explanation is that if the patient's viral load is less than 1000 copies/cell, the CD4 count rise, and immune recovery is good. Therefore, the patient was free from opportunistic infection and had a good intake, absorption, and appetite.

Participants who were farmers were less likely to develop overnutrition compared to drivers and housewives. This finding is not consistent and significant in association with other studies reported from different countries^16^,^20^,^22^,^37^. The possible reason might be the habit of feeding, availability, accessibility, and utilization of food, nature of their work, and physical activity.

Finally, the participant who has been taking prophylaxis therapy statically significant associated factors with overnutrition This finding is not aligned and significant association with reports of studies conducted in different countries^5^,^15^,^16^,^22^. This might be because patients have been taking prophylaxis therapy, they will be free from an opportunistic infection, and the patient also had good food intake, absorption, and free mal-absorption syndrome.

## Conclusions

In this study, one-fifth of Human Immunodeficiency Virus/Acquired Immunodeficiency Virus patients had over nutrition. In this study, the magnitude of overnutrition is high associated with a viral load of fewer than 1000 copies/cell, age greater than 45, and having taken prophylaxis therapy. Therefore, educating about the use of lifestyle change, regular monitoring of weight, regular nutritional assessment, and intervention of the existed problems like doing regular exercise is highly recommended.

## Limitations of the study

Since the study design is cross-sectional cause and effect relationship cannot be established. Moreover, the physical activity status of patients was obtained from self-report, and recall bias may have influenced the results.

## Viability of data and material

Data will be available upon reasonable request of the corresponding author.
